# Adult-onset linear discoid lupus erythematosus on the forehead mimicking en coup de sabre: a case report

**DOI:** 10.1186/s13256-019-2249-7

**Published:** 2019-11-27

**Authors:** Sasipaka Sindhusen, Kumutnart Chanprapaph, Suthinee Rutnin

**Affiliations:** Division of Dermatology, Department of Medicine, Ramathibodi Hospital, Mahidol University, 270 Rama VI Road, Ratchatewi, Bangkok, 10400 Thailand

**Keywords:** Linear lupus erythematosus, Discoid lupus erythematosus, En coup de sabre, Linear morphea, Blaschko line

## Abstract

**Background:**

Linear cutaneous lupus erythematosus (LE) is an unusual form of LE-specific cutaneous condition, occurring in children and young adults. Due to its rarity, the diagnosis of linear cutaneous LE can be difficult and facial lesions can resemble linear morphea or en coup de sabre. Differential diagnosis of similar conditions along the lines of Blaschko must be differentiated from linear LE.

**Case presentation:**

We report a case of linear discoid LE on the forehead of an adult female Thai patient mimicking en coup de sabre. The dermatoscopy, histopathology and direct immunofluorescence findings were consistent with chronic cutaneous LE.

**Conclusions:**

As this patient demonstrated classic dermatoscopic features of LE, we emphasized that the diagnosis of linear cutaneous LE can be made by dermatoscopy. This is particularly beneficial for young self-conscious patients with facial lesions that are reluctant to perform skin biopsy.

## Background

Lupus erythematosus (LE) is an autoimmune connective tissue disease characterized by a heterogenous spectrum of skin and internal organ manifestations. Linear discoid lupus erythematosus (LDLE) is a rare variant of DLE characterized by linear erythematous plaques along the lines of Blaschko. Differential diagnosis must be considered with other linear conditions. Due to its rarity, histopathological examination is mainly required to establish the definitive diagnosis. According to Lallas *et al.* [1], dermatoscopy may enhance the clinical diagnosis of DLE especially in the patient who cannot perform a skin biopsy. We hereby report DLE in an adult patient presenting with a linear variant on the forehead clinically mimicking en coup de sabre. We emphasize that the characteristic dermatoscopic findings including structureless whitish areas, follicular keratotic plugs, telangiectatic vessels and brown to grayish pigmentation are of great value in differentiating DLE from other linear conditions distributed along the lines of Blaschko.

## Case presentation

**Fig. 1:**
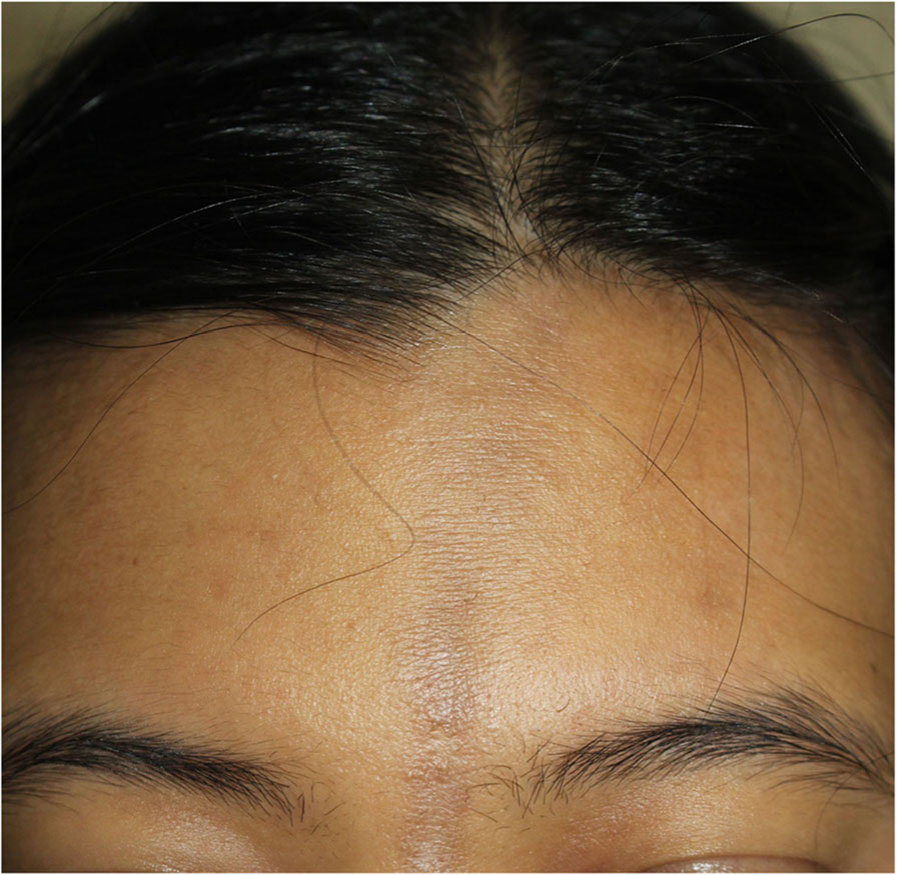
Linear erythematous to purplish atrophic patch on the forehead

**Fig. 2:**
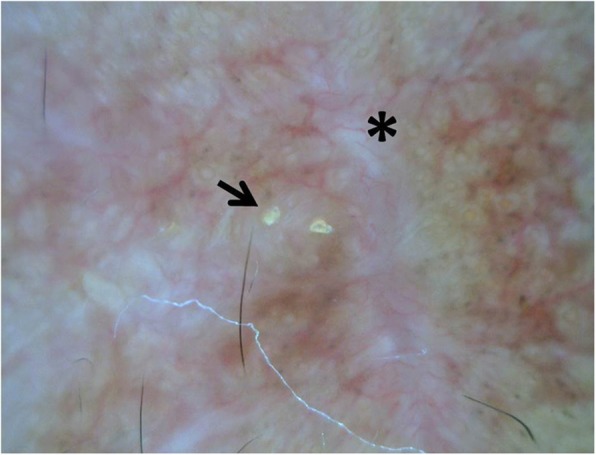
Dermatoscopy demonstrated structureless whitish areas (asterisk), follicular keratotic plugs (arrow), telangiectatic vessels and brown to grayish pigmentation (original magnification × 50)

**Fig. 3:**
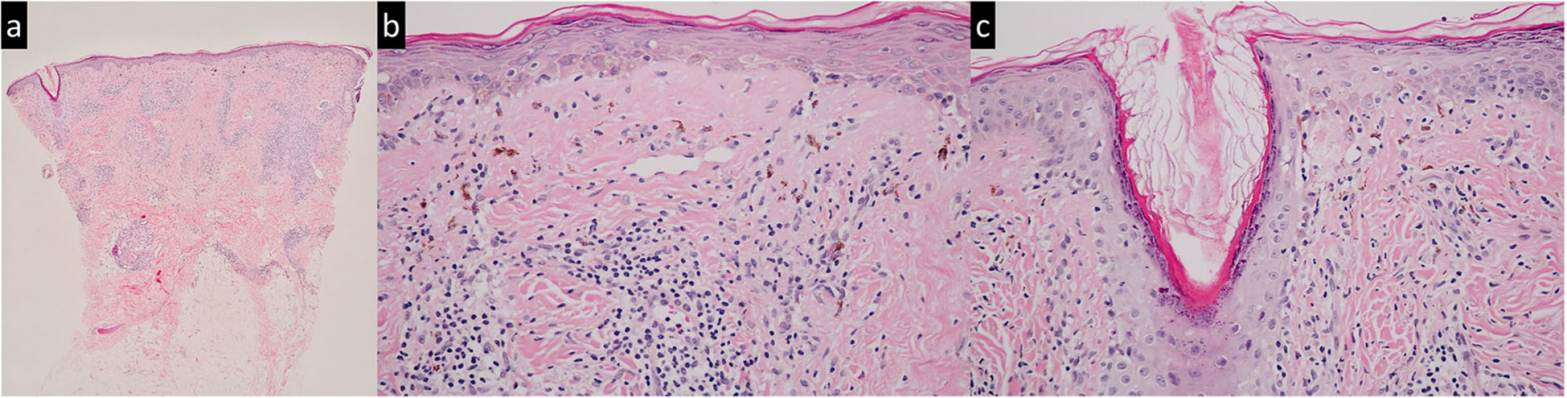
Histopathology of linear discoid lupus erythematosus (**a**) superficial and deep perivascular and periadnexal lymphocytic infiltrate (haematoxylin and eosin stain, original magnification × 40) (**b**) epidermal atrophy, hydropic degeneration of the basal cell layer, numerous melanophages, and telangiectasia (haematoxylin and eosin stain, original magnification × 400) (**c**) follicular interface change with follicular hyperkeratosis (haematoxylin and eosin stain, original magnification × 400)

**Fig. 4:**
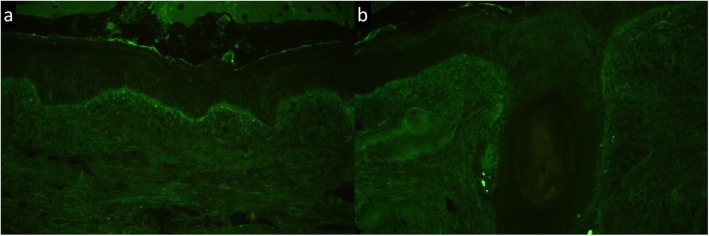
Direct immunofluorescence showed homogenous granular deposition of IgM and few cytoid bodies along the basement membrane (**a**) and follicular epithelium (**b**) (original magnification × 400)

A 28-year-old Thai office worker from Bangkok presented with a 3-month history of an asymptomatic linear erythematous to purplish atrophic patch on the forehead following the lines of Blaschko (Fig. 1). She had no history of trauma preceding the lesions or photosensitivity. She had no underlying disease or family history of malignancy or other autoimmune connective tissue diseases. She is an only daughter living with her family that belongs to middle socioeconomic class. Her menstrual cycle was normal. She had no history of smoking or alcoholic consumption. She was previously treated at a provincial hospital with mometasone furoate cream 0.1% once daily for 3 weeks but the lesion did not improve. On physical examination, her temperature was 36.7 °C, blood pressure 119/83 mmHg, pulse rate 100/minute and respiratory rate 20/minute. Cardiovascular and respiratory assessments were normal. Neurological examination showed all intact cranial nerves. Motor and sensory examinations were within normal limits. Other dermatological examinations were unremarkable including mucous membranes, scalp, hair and nails. The initial diagnosis was en coup de sabre. Dermatoscopic evaluation demonstrated structureless whitish areas, follicular keratotic plugs, telangiectatic vessels and brown to grayish pigmentation (Fig. 2), which are reminiscent of DLE. Therefore, after obtaining inform consent, a skin biopsy specimen from the forehead was performed and revealed epidermal atrophy, follicular plugging, and hydropic degeneration of the basal cell layer. Superficial and deep perivascular and periadnexal lymphocytic infiltrate with numerous melanophages, telangiectasia, and dermal fibrosis (Fig. 3). Direct immunofluorescence (DIF) of a lesional skin revealed homogeneous granular deposition of immunoglobulin M (IgM) along the dermoepidermal junction and follicular epithelium with few cytoid bodies of IgM (Fig. 4). Her complete blood count (CBC) showed hemoglobin (Hb) level of 13.4 g/dL, white blood cell (WBC) count of 6.3 × 10^9^/L composing of 69% neutrophils, 26% lymphocytes, and 5% monocytes, and platelet count of 233 × 10^9^/L. Liver function test (LFT) revealed total and direct bilirubin levels of 0.4 mg/dL and 0.2 mg/dL, respectively, aspartate aminotransferase (AST) 18 IU/L, alanine aminotransferase (ALT) 11 IU/L, alkaline phosphatase (ALP) 44 IU/L, and gamma-glutamyl transferase (GGT) 13 IU/L. She had negative hepatitis B virus surface antigen (HBsAg) and anti-hepatitis C virus (anti-HCV) in her serum. Renal function test showed blood urea nitrogen (BUN) of 9 mg/dL and creatinine of 0.6 mg/dL. No proteinuria was observed and there were 0–1 white blood cells and red blood cells /high power field in urinalysis. Antinuclear antibody titer was negative. Based on the clinical and dermatoscopic examinations, histological and DIF results, the diagnosis of linear DLE was made. Tacrolimus ointment 0.1% was applied twice daily on the lesion alongside with hydroxychloroquine (200 mg/day). Strict sun protection was also advised. At 6-month follow-up, the lesions showed significant improvement and resolved with atrophic patch.

## Discussion

We report a case of linear discoid LE on the forehead of an adult female patient mimicking en coup de sabre. To the best of our knowledge, this is the first case emphasizing on the characteristic dermatoscopic findings of LDLE on the forehead which are important information in differentiating DLE from other linear conditions distributed along the lines of Blaschko.

Cutaneous LE (CLE) presenting with linear configuration is exceeding rare. A linear variant of CLE was first described in 1978 by Umbert and Winklemann [2] as a cutaneous mixed or overlap syndrome between linear scleroderma and DLE. Later in 1998, Abe *et al.* [3] reported 2 cases of linear childhood CLE following the line of Blashko and proposed the term linear cutaneous lupus erythematosus (LCLE) to described this rare variant of CLE.

According to the literature review, a total of 102 cases diagnosed with LCLE have been reported. The most common pathological diagnosis of CLE in the linear distribution was discoid lupus erythematosus (DLE) (39.2%), followed by lupus erythematosus panniculitis (LEP) (21.6%), lupus erythematosus tumid (LET) (2.9%), subacute lupus erythematosus (SCLE) (2%) and bullous lesions of SLE (1%) [4–13]. LDLE mainly occurs in children and young adults. The mean age onset was 22.1 years (1–68 years). Unlike the female predominance typically seen in SLE patients, no gender predilection was noted in LCLE with female to male ratio of 1.3:1. The typical clinical manifestations were single or multiple linear asymptomatic erythematous plaques along the lines of Blaschko. Lesions developed most commonly on head followed by neck, trunk, and extremities. There have been only 9 reported cases of adult-onset linear CLE on the forehead which could be mistaken for en coup de sabre (Table 1). LE-associated serology such as the antinuclear antibodies are mostly negative. Photosensitivity and progression to SLE are rarely observed [7, 9, 10].

**Table 1:** Summary of previous reported cases of adult-onset linear cutaneous lupus erythematosus on the forehead [7]

No.	Sex	Age of onset	Duration	Location	Pathological diagnosis	DIF	ANA	Other autoantibodies	Progression to SLE	Treatment
1	F	29 y	3 y	Forehead	CLE	+Granular IgM	–	NA	No	TC, HCQ
2	F	21 y	Few m	Forehead, scalp	LET	NA	9 y later: +ANA	9 y later: +anti-dsDNA, +RF	Yes	ILC 9 y later: IC, OC, CTX, HCQ
3	M	49 y	1 y	Forehead, inner eye angle, right lateral aspect of nose	CCLE	+Band-like IgG, IgM, IgA, C3	–	NA	No	HCQ
4	M	21 y	1 y	Forehead, left supraorbital and infraorbital area, left angle of mouth	CLE	+	–	+Anti-SSA, +anti-SSB	No	HCQ, TC, TCI
5	M	33 y	1 y	Forehead	CCLE, Scl	+Granular IgM	–	-Anti-RNP, -anti-NCS, -anti-dsDNA	No	CQ, TC, TCI
6	M	33 y	2 m	Forehead, left eyelid, nose	CLE	NA	–	NA	No	HCQ, TC
7	F	24 y	2 y	Forehead, scalp	LEP	+Granular IgG	+ANA	+Anti-SSA -anti-dsDNA, -anti-ssDNA, -anti-Sm, -anti-SSB	No	OC
8	M	42 y	1 y	Forehead, right upper eyelid	LET	+Granular IgM, C3	–	NA	No	OC, HCQ
9	F	24 y	8 y	Forehead, scalp, left cheek	DLE, Scl	NA	NA	NA	No	OC, CQ, TC, TCI

Abbreviations: ANA, anti-nuclear antibodies; anti-dsDNA, anti-double stranded DNA; anti-NCS, antinucleosome; anti-RNP, anti-ribonucleoprotein; anti-Sm, anti-Smith; anti-ssDNA, anti-single stranded DNA; C3, complement C3; CCLE, chronic cutaneous lupus erythematosus; CLE, cutaneous lupus erythematosus; CQ, chloroquine; CTX, cyclophosphamide; DIF, direct immunofluorescence; DLE, discoid lupus erythematosus; F, female; HCQ, hydroxychloroquine; IC, Intravenous corticosteroid; IgA, immunoglobulin A; IgG, immunoglobulin G; IgM, immunoglobulin M; ILC, intralesional injection of corticosteroid; LEP, lupus erythematosus panniculitis; LET, lupus erythematosus tumid; M, male; m, month; NA, Not available; No., number; OC, oral corticosteroid; RF, rheumatoid factor; Scl, scleroderma; SLE, systemic lupus erythematosus; TC, topical corticosteroid; TCI, topical calcineurin inhibitors; y, year

The exact pathogenesis of LCLE is unknown. Alfred Blaschko first described the lines of Blaschko in 1901 and represent the developmental growth pattern of the embryonic ectodermal cells. In the linear variant of CLE, genetic mosaicism/epigenetic modification of keratinocytes and the immune system in the lines of Blaschko may play a potential role in development of this specific CLE variant [7, 14]. The keratinocytes in the lines of Blaschko triggered by trauma, irritation, or ultraviolet light may express antigens and introduce a wide range of stimuli crucial for the development of CLE. Keratinocyte apoptosis has also been indicated as a key event in initiating CLE through various apoptotic pathway such as p53, tumor necrosis factor alpha (TNF-α), and Fas/Fas Ligand (FasL). However, it remains a speculation whether these genetically variant keratinocytes are indeed lacking proteins essential for regulation of apoptosis, are there immunological distinct showing aberrant major histocompatibility complex (MHC) expression, or are there relaxing abnormal aberrant cytokines [7, 14].

The differential diagnosis of LCLE includes other acquired inflammatory skin diseases distributed along the lines of Blaschko, such as linear morphea, linear lichen planus, lichen striatus, linear psoriasis, linear lichen sclerosus and linear granuloma annulare [10].

Although, the final diagnosis of CLE was achieved by histopathological examination, dermatoscopic findings also play an important role to identify the accurate diagnosis. Lallas *et al.* described the dermatoscopic criteria of DLE located on the face, trunk and extremities, and correlated them to the underlying histopathology. Perifollicular whitish halo, follicular keratotic plugs and telangiectasia were the most common dematoscopic findings in DLE [1].

In our patient, some dermatoscopic features correspond well to the histopathological findings. Dermatoscopic findings showed follicular keratotic plugs, telangiectatic vessels, pigmentation, and structureless whitish areas, representing histopathologic features of follicular hyperkearatosis, telangiectasia, pigmentary incontinence, and diffuse dermal fibrosis, respectively. In addition to dematoscopic and histopathologic diagnosis, direct immunofluorescence (DIF) of lesional skin is also the useful adjunction for diagnosing LDLE. These findings are granular depositions of IgG, IgM, IgA, and/or C3 along the dermoepidermal junction and periadnexal structure. Nonetheless, a negative DIF result does not exclude the diagnosis [10].

In linear DLE, topical high-potency corticosteroids and photoprotection are the mainstay of therapy [15]. Topical calcineurin inhibitors are reserved for long-term maintenance [16]. In cases presenting with widespread lesions unresponsive to topical therapy, antimalarials especially hydroxychloroquine may be considered as the first-line drug. Other systemic treatments including oral corticosteroids, dapsone, thalidomide, mycophenolate mofeti, azathioprine and retinoids have been reported to be benificial [16].

## Conclusions

We hereby reported a case of adult-onset LDLE presented with linear erythematous to purplish atrophic patch on the forehead along the lines of Blaschko masquerading en coup de sabre. We would like to encourage physicians to use dermatoscopy to establish a clinical diagnosis, particularly in young self-conscious patients with facial lesions and are hesitant to perform skin biopsy.

## Data Availability

Please contact the authors for data requests.
